# The Effect of Acute Intense Exercise on Activity of Antioxidant Enzymes in Smokers and Non-Smokers

**DOI:** 10.3390/biom11020171

**Published:** 2021-01-27

**Authors:** Hadi Nobari, Hamzeh Abdi Nejad, Mehdi Kargarfard, Soghra Mohseni, Katsuhiko Suzuki, José Carmelo Adsuar, Jorge Pérez-Gómez

**Affiliations:** 1 Department of Exercise Physiology, Faculty of Sport Sciences, University of Isfahan, Isfahan 81746-7344, Iran; m.kargarfard@spr.ui.ac.ir; 2 HEME Research Group, Faculty of Sport Sciences, University of Extremadura, 10003 Cáceres, Spain; carmelo.adsuar@gmail.com (J.C.A.); jorgepg100@gmail.com (J.P.-G.); 3 Department of Exercise Physiology, University of Guilan, Rasht 4199843653, Iran; hamzehabdi15@gmail.com; 4 Department of Exercise Physiology, Faculty of Sport Sciences, Kharazmi University, Tehran 14911-15719, Iran; tarbeyat88@gmail.com; 5 Faculty of Sport Sciences, Waseda University, Saitama 359-1192, Japan

**Keywords:** exhaustive exercise, oxidative stress, regular physical activity, saliva, peroxidase, catalase, superoxide dismutase

## Abstract

Acute intense exercise causes significant oxidative stress and consequently an increase in total antioxidant capacity; however, the mechanisms and combined effects of intense exercise and smoking on oxidative stress among active and non-active smokers are not clear. The aim of this study was to investigate the effect of acute intense exercise on antioxidant enzyme activity responses in active and non-active individuals exposed to cigarette smoke. The study included 40 subjects who were equally classified as: smokers that did exercise (SE), smokers that did not do exercise (SnE), non-smokers that did exercise (NSE), and non-smokers that did not do exercise (NSnE). The adjusted Astrand test was used to exhaust the subjects. Salivary enzymes of peroxidase (POX), catalase (CAT), and superoxide dismutase (SOD) were measured, by spectrophotometry methods, at 3 different time points: pre-test (TP1), post-test (TP2), and one hour after finishing the test (TP3). Significant (*p* < 0.05) group x time interactions were found for the three enzymes. Salivary POX, CAT and SOD increased in all groups from TP1 to TP2 and decreased from TP2 to TP3. Only the NSE showed a significant difference between TP1 to TP3 in POX and SOD by +0.011 ± 0.007 and +0.075 ± 0.020 (U/mL), respectively. The NSE showed significantly higher activity of POX, CAT and SOD in TP2 compared to the other groups. Furthermore, NSE and NSnE had higher activity of POX, CAT and SOD in TP1 and TP3 (*p* < 0.05) compared with SE and SnE. Only in the NSnE, were no differences observed in CAT compared with SE and SnE in TP3. These results showed that the antioxidant activity at rest and in the recovery time after the acute intense exercise was lower in SE and SnE compared with NSE and NSnE, suggesting that smoking habit may reduce the ameliorating effect of regular physical activity on acute exercise-induced oxidative stress.

## 1. Introduction

Smoking is one of the most serious global health problems [1]. Cigarette smoke contains dozens of complex chemicals that can cause some important diseases such as lung cancer, asthma, and chronic obstructive pulmonary disease [2,3]. Moreover, cardiovascular and respiratory problems may also be caused or exacerbated by smoking. Research has shown that cigarette smoke contains more than 4000 types of chemicals [3]. According to the World Health Organization smoking produces more than 8 million deaths worldwide. There are 1.1 billion smokers in the world with 1.2 million adults who are involuntarily exposed to cigarette smoke [4]. 

Smoking is a negative factor that can increase free radical generation or decrease the activity of antioxidant enzymes in the body. Lipid peroxidation is the oxidative degradation of lipids. It is the process in which free radicals “steal” electrons from the lipids in cell membranes, resulting in the generation of reactive oxygen species (ROS) in quantities that exceed antioxidant capacity, and cell damage which eventually may cause cell death [5,6]. Overproduction of ROS cause a wide range of cellular damage, such as enzyme inactivation, lipid peroxidation, and lipoprotein oxidation [7]. Free radicals may be involved in the pathogenesis of cardiovascular disease and cancer. Cigarette smoke can alter the metabolism of trace elements which are required at low concentrations such as essential components of antioxidant enzymes like copper and zinc as cofactors of superoxide dismutase (SOD) and iron for catalase (CAT). Therefore, cigarette smoke can reduce the activities of antioxidant enzymes [8].

Research has shown that high-intensity and/or prolonged exercise may cause muscle tissue damage through the reactions of free radicals. The increase in free radicals, following muscle injury, has been suggested to cause secondary muscle damage directly or indirectly, via oxidative damage of biomolecules or induction of inflammatory cytokines [9,10]. Physical fitness levels play a role in the overproduction of ROS and changes in antioxidant enzymes and can lead to different degrees of oxidative damage [9,11]. Hence, in physical activity antioxidant capacity is a measure used as an oxidative stress indicator, and it increases to neutralize ROS and protect body cells. Cells have developed different antioxidant systems and various antioxidant enzymes to defend themselves against free radical attacks. SOD, the first line of defense against oxygen-derived free radicals, catalyzes the dismutation of the superoxide anion into hydrogen peroxide (H_2_O_2_). The H_2_O_2_ can be transformed into H_2_O and O_2_ by CAT [12]. ROS can damage large molecules such as membrane lipids, nucleic acids and cellular proteins [13]. Studies in humans have also confirmed that exercise may be very beneficial in the defense and prevention of smoking-dependent oxidative stress and inflammation [14].

During exercise, the inner membrane of the mitochondria is an important source of ROS, and the intensity or volume of exercise determines the activity of free radical overproduction that can lead to different degrees of oxidative damage [15,16]. Moreover, smokers face reduced antioxidant capacity and have higher oxidative damage than non-smokers [17], due to deficient retaining of the balance of ROS in the body by antioxidant agents during acute intense exercise. The present study investigated the responses of salivary peroxidase (POX), CAT, and SOD activities after acute intense exercise in four groups: non-smokers that did not exercise (NSnE), non-smokers that did exercise (NSE), smokers that did not exercise (SnE), and smokers that did exercise (SE). Thus, saliva is secreted as a biological fluid from glands such as the parathyroid, submandibular, and sublingual, and because it is non-invasive and cheaper than other evaluation methods, it can be widely used in clinical studies [18]. Previous studies have reported that the responses of salivary anti-oxidative markers, including POX activity (9%) and the percentage of radical scavenging activity (7%) after a single exercise session were weaker in water pipe tobacco smokers than non-smokers [19]. To the best of our knowledge, no data are available concerning the comparison of salivary anti-oxidative enzyme responses of the above groups to acute intense exercise. Ultimately, the present study investigated the effects of high-intensity exercise and/or smoking on antioxidant enzymes.

## 2. Materials and Methods

### 2.1. Participants

**Figure 1:**
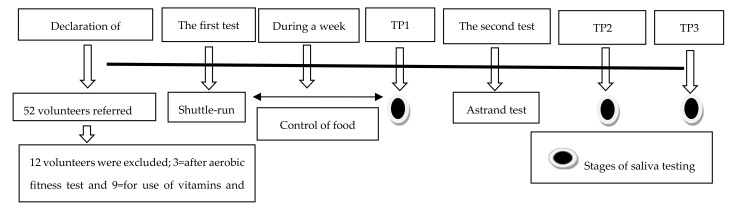
Diagram of the research outline. TP1 = time point from Pre-test, TP2 = time point immediately after the test, TP3 = time point one hour after the test.

A total of forty young men (mean ± standard deviation); age, 22.93 ± 1.76 years; height, 1.77 ± 0.40 m; weight, 72.10 ± 4.91 kg; body fat, 13.86 ± 2.09%; VO_2max_, 48.40 ± 8.06 mL.kg^−1^.min^−1^) were divided into four groups: NSnE (*n* = 10), NSE (*n* = 10), SnE (*n* = 10) and SE (*n* = 10). The inclusion criteria for NSnE were: no regular exercise nor smoking in the previous six months, the criteria for NSE were: regular physical activity, 3–4 sessions per week, and no smoking in the previous six months. For SnE the criteria were being smokers over the previous six months, having smoked between 10–12 cigarettes daily for about 3–5 years and having performed no regular exercise, and finally, the SE who had regular exercise (3–4 sessions per week) and smoked like the previous group. Additional inclusion criteria for all groups were: (1) Subjects should not have an infection in the mouth; (2) No vitamin intake in the previous six months (nine were excluded from the study due to supplement use in the previous six months; (3) To guarantee groups with similar physical fitness levels, subjects with very high/low VO_2max_ were excluded (Figure 1). All the subjects signed and accepted the informed consent according to the recommendations of the Helsinki Declaration for Human Research, and the Ethics Committee of the University of Isfahan approved the study.

### 2.2. Experimental Approach to the Problem

This is semi-experimental research that has been done with pre- and post-test with repeated measurements. Before starting height, weight, and body fat percentage (BF%) were assessed, the thickness of the subcutaneous fat layer of the chest, abdomen, and thigh was measured using a calibrated caliper (Lafayette Instrument Company, Lafayette, IN, USA) with an accuracy of 0.1 mm, and then estimated using the numbers with the Jackson and Pollock equation [20,21]. Body composition and anthropometric measurements were performed in the morning [22,23]. In the next stage, subjects began the warm-up under an instructor’s supervision for 10 min, then performed a shuttle run test to assess aerobic fitness according to guidelines [24] to finalize the groups and VO_2max_ measurement (Table 1). Three days later, a protocol and training session on how to rinse and salivate was held in the form of videos, oral explanations and written dietary advice. All subjects had to eat lunch three hours earlier and a list of citrus and antioxidant-containing foods was set individually for each group. They were advised not to do exercise for 48 h before the experiment, and the smoker groups did not smoke for the previous 24 h; subjects received oral and written information about food intake prior to protocol implementation, and three days before the test, subjects had begun to record and send in daily food diaries.

One week later, subjects arrived at the test site from 15:00 p.m., and the temperature was between 23 and 26 °C. Subjects were asked to eat their food in a homogeneous manner, and washed their mouths with distilled water and brushing before collecting saliva, to remove excess oral debris, and then collected saliva in sterile tubes for 5 min [16]. Samples were collected at three different time points (TP): TP1 (baseline), TP2 (at the end), and TP3 (1 h after the end). The samples were transferred to the laboratory immediately after each collection and centrifuged there. Each subject performed the second test (Astrand test) after a 10-min warm-up under the guidance of the instructor.

### 2.3. Food Control

With the help of a nutritionist, a list of approximate calories from all native foods (exclusively Iranian) was prepared and distributed to the subjects. A briefing session was held on how to comply with the calorie intake instructions. After the briefing session until the day of protocol execution in the week, and during the last day leading up to the protocol, the total calorie intake was calculated using Nutrition 4 version 3.5.2 software made in Iran [25]. Every three days, participants submitted their nutrition notes to the researcher through the WhatsApp application, immediately calorie counts and antioxidant intake were calculated, and if necessary, recommendations were made. Suggested values were presented daily to the participants in a sheet that for vitamin C, was on average 80 mg, and for vitamin E was 16 mg [26]. Daily reports by subjects showed that there was no significant difference among the four groups in their consumption of nutrients and vitamins related to antioxidant effects (Table 2).

### 2.4. Procedures

#### 2.4.1. Acute Intense Exercise

In this study, the Astrand test was considered as an acute intense exercise. For this purpose, the Italian Technogym treadmill was used. At the beginning of the test, the subject first started running on an incline of zero and speed at 8.05 km/h for three minutes. After 3 min the incline was set to 2.5% for 2 min and the other steps were added every two minutes with the same time and slope until the participant was not able to continue running [27,28]. 

#### 2.4.2. Monitoring Internal Load

**Figure 2:**
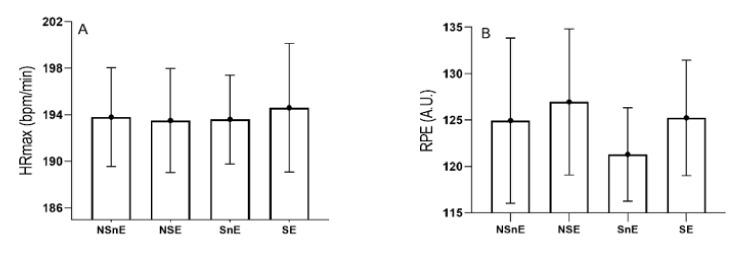
Descriptive Mean and SD of recorded internal load of the acute intense exercise using (**A**) HRmax and (**B**) RPE for each group separately. NSnE = non-smokers that did not exercise; NSE = non-smokers that did exercise; SnE = smokers that did not exercise; SE = smokers that did exercise; RPE = Ratings of Perceived Exertion; A.U. =Arbitrary unit.

The internal monitoring load, using subjects’ heart rates, was measured with the polar watch AXN500. We also used the ratings of perceived exhaustion (RPE) for workload monitoring. The 10-point RPE or Borg scale is a valid indicator of internal workload [20,29]. After finishing the Astrand test, the subjects were asked to indicate their perception of the test load. Once the load was indicated during the test, it was multiplied to calculate the workload applied to each subject during the protocol [20,30] (Figure 2A,B).

#### 2.4.3. Chemical Resources

All of chemicals used were purchased from Merck. Analytical grade of these chemicals were analyzed according to the manufacturer’s instructions without any changes. These processes were performed in the University Biochemistry Laboratory and their pH was checked twice. 

#### 2.4.4. Saliva Sampling

**Figure 3:**
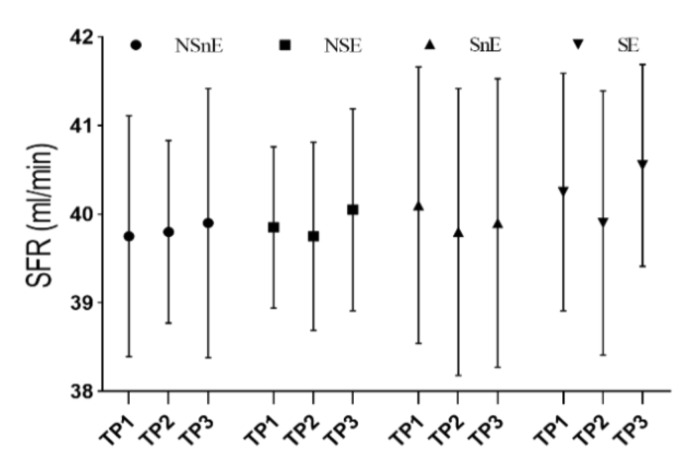
Change in salivary flow rate for the four groups, and for each time point. TP1 = time point from Pre-test, TP2 = time point immediately after the test, TP3 = time point one hour after the test; NSnE = non-smokers that did not exercise; NSE = non-smokers that did exercise; SnE = smokers that did not exercise; SE = smokers that did exercise; SFR= Salivary Flow Rate.

The amount of saliva collected for a set time (5 min) was measured and then the unstimulated salivary flow rate was calculated (Figure 3). Saliva samples were centrifuged at 7500 rpm for 10 min in a temperature-controlled room 4 °C and placed in a freezer at −70 °C until analysis to enable enzyme activity measurement at a time.

#### 2.4.5. POX Activity Measurement

To evaluate the activity of the POX enzyme, a 4-amino-antipyrine substrate was used in the presence of hydrogen peroxide. The sample contained 425 μL of 4-amino-antipyrine +425 μL of hydrogen peroxide and 150 μL of saliva. The blank tube (control) also contains 425 μL of 4-amino-pyrine, 425 μL of hydrogen peroxide and 150 μL of 0.2 μm phosphate buffer with pH equal to 7.0. Samples were added to the substrate after reaching the standard temperature, and the enzyme reaction was studied using a spectrophotometer (Pharmacia manufactured by Biotech Model 3000) at 510 nm and absorption changes were recorded over time. Finally, the data were divided by the number 6.58 and the activity of POX was calculated [31].

#### 2.4.6. CAT Activity Measurement

To measure the activity of CAT, 50 mM phosphate buffer (pH = 7.0) were combined with 10 mM hydrogen peroxide and then two cuvettes made of quartz with a volume of 1 cm were selected. With cuvette blank, 500 μL of phosphate buffer and hydrogen peroxide were combined and 250 μL of phosphate buffer, hydrogen peroxide, and 50 μL of saliva were added to another cuvette, using a spectrophotometer (Pharmacia manufactured by Biotech Model 3000) in the form of a Kinetik at 240 nm for 1 min and recorded in 5-s intervals and finally, the numbers obtained were divided by 39.4 and the activity of CAT enzyme was calculated [32]. One unit of the enzyme CAT was the amount of decomposition of one μmol of hydrogen peroxidase per minute at a temperature of 25 °C and pH = 7 to oxygen and water, using 50 mM hydrogen peroxide Measurements of enzyme activity were used according to Aebi.

#### 2.4.7. SOD Activity Measurement

The total activity of SOD was assayed using the Gianopulitis and Reice method of measuring its ability to prevent the reduction of nitro blue tetrazolium (NBT) photochemical reduction. SOD enzyme activity was measured using a spectrophotometric method. The test mixture was 0.5 mL of reaction solution (EDTA 0.1 mM, 50 mM phosphate buffer, 75 mM NBT and 0.21 mM Riboflavin) with one mL of saliva. The cuvettes containing the reaction solution were exposed to the fluorescence beam (20 watts of fluorescence) for 30 min and then read for the absorbance of the samples and control in a 560 nm wavelength belt against Blank. Finally, the amount of SOD was determined using the following formula: (OD test-OD Blank/OD Blank) × 100 [33]. Each SOD unit is the amount of enzyme required for 50% inhibitory photochemical restoration of NBT under test conditions.

### 2.5. Statistical Analysis

Homogeneity of variables in the research groups was determined with the Leven test and the data were normalized using the Shapiro–Wilk test. Then, inferential tests were executed. Changes between the three time points were assessed using a repeated-measures analysis of variance (ANOVA), followed by the Bonferroni post hoc test for pairwise comparisons. Besides, a one-way ANOVA was applied to compare the different salivary activities of variables, by groups and time points; followed by the Bonferroni post hoc test for pairwise comparisons. Data analysis was performed using SPSS software (23.0; IBM SPSS Inc., Chicago, IL, USA) and the significance level was set at *p* < 0.05 at all stages. Charts were drawn with GraphPad Prism 8.0.1.

## 3. Results

There were no significant (*F* (2, 0.900) *p* > 0.05) main effects of time or group by time interaction (*F* (6, 0.286) *p* > 0.05) for changes in salivary flow rate (Figure 3).

**Figure 4:**
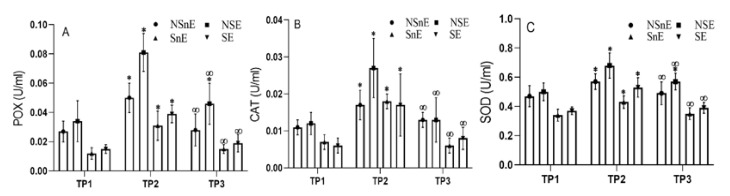
Change in (**A**) POX = peroxidase, (**B**) CAT = catalase, and (**C**) SOD = superoxide dismutase for each time point. * Represents a statistically significant difference compared to TP1 (*p* < 0.05); ∞ Represents a statistically significant difference compared to TP2 (*p* < 0.05); TP1 = time point from Pre-test, TP2 = time point immediately after the test, TP3 = time point one hour after the test; NSnE = non-smokers that did not exercise; NSE = non-smokers that did exercise; SnE = smokers that did not exercise; SE = smokers that did exercise.

Salivary activities of POX demonstrated main effects of time (*F* (1, 263.49) *p* ≤ 0.001), group x time effect (*F* (3, 11.85) *p* ≤ 0.0001), and group effect (*F* (3, 35.095) *p* ≤ 0.001). Post hoc tests using the Bonferroni correction revealed a significant increase in POX activity in all groups between TP1 and TP2; NSnE: (mean diff = +0.023 ± 0.009; *p* ≤ 0.001), NSE: (mean diff = +0.046 ± 0.008; *p* ≤ 0.001), SNE: (mean diff = +0.019 ± 0.008; *p* ≤ 0.001) and SE: (mean diff = +0.024 ± 0.006; *p* ≤ 0.001), respectively. Then, this variable decreased in all groups between TP2 to TP3; NSnE: (mean diff = −0.022 ± 0.013; *p* = 0.002), NSE: (mean diff = −0.035 ± 0.009; *p* ≤ 0.001), SnE: (mean diff = −0.016 ± 0.008; *p* = 0.001) and SE: (mean diff = −0.020 ± 0.005; *p* ≤ 0.001). There was only a significant difference between TP1 to TP3 in NSE (+0.011 ± 0.007; *p* = 0.002) (Figure 4A). Likewise, this variable was also analyzed by one-way ANOVA with comparison among three time points in between-group differences, and it was demonstrated that there was a difference in POX activities which were higher compared to the NSE with other groups in TP1; NSE vs. NSnE in TP3, and ultimately NSnE vs. both smoker groups in TP3 (*p* < 0.05) (Table 3).

Salivary activities of CAT demonstrated main effects of time (*F* (2, 135.79) *p* ≤ 0.001), group x time effect (*F* (6, 3.33) *p* = 0.006), and group effect (*F* (3, 13.95) *p* ≤ 0.001). Furthermore, within groups, time differences showed that a significant increase in CAT activity occurred in all groups between TP1 and TP2; NSnE: (mean diff = +0.006 ± 0.006; *p* = 0.016), NSE: (mean diff = +0.015 ± 0.007; *p* = 0.001), SnE: (mean diff = +0.011 ± 0.003; *p* = 0.001) and SE: (mean diff = +0.010 ± 0.004; *p* = 0.001), respectively, and then these variables decreased in all groups between TP2 and TP3; NSnE: (mean diff = −0.008 ± 0.004; *p* = 0.001), NSE: (mean diff = −0.013 ± 0.007; *p* = 0.001), SnE: (mean diff = −0.012 ± 0.003; *p* = 0.010) and SE: (mean diff = −0.009 ± 0.002; *p* = 0.001). However, there was no significant difference in any group between TP1 and TP3 (Figure 4B). The between-group differences demonstrated that there was a significant difference in CAT with higher values in NSE and NSnE compared to SnE and SE in TP1; as well as NSE compared to the other groups in TP2 and with SnE and SE in TP3 (*p* < 0.05) (Table 3).

Salivary activities of SOD demonstrated main effects of time (*F* (1.33, 108.02) *p* ≤ 0.001), group x time effect (*F* (3.99, 3.46) *p* = 0.015), and group effect (*F* (3, 36.72) *p* ≤ 0.001). The results revealed that a significant increase in SOD activity occurred in all groups between the TP1-TP2; NSnE: (mean diff = +0.099 ± 0.04; *p* ≤ 0.001), NSE: (mean diff = +0.178 ± 0.08; *p* ≤ 0.001), SnE: (mean diff = +0.088± 0.05; *p* = 0.002) and SE: (mean diff = +0.161 ± 0.07; *p* = 0.001), and then these variables decreased in all groups between TP2 and TP3; NSnE: (mean diff = −0.078 ± 0.05; *p* = 0.002), NSE: (mean diff = −0.103 ± 0.08; *p* = 0.010), SNE: (mean diff = −0.072± 0.07; *p* = 0.045) and SE: (mean diff = −0.136 ± 0.08; *p* = 0.001). Only a significant difference was observed between TP1 and TP3 time points in NSE (0.075 ± 0.02; *p* = 0.001), and there was no significant difference in other groups (Figure 4C). Moreover, this variable in the results of between-group demonstrated that there was a significant difference in the resting activities, SOD values in the groups NSE and NSnE were higher than those of SnE and SE in TP1; as well as NSE compared with the other groups in TP2, but SnE decreased in comparison with other groups in TP2. Finally, the NSE and NSnE maintained higher activities of this variable in TP3 than the other two groups, and the NSE had higher activities than the NSnE (*p* < 0.05) (Table 3).

## 4. Discussion

The results of the current study demonstrated that acute intense exercise increased antioxidant activity, but smoking decreased resting activity of antioxidant enzymes, whereas immediately after exhaustion, SOD and CAT activities in SE and SnE were lower than NSE. The activity of all enzymes in TP3 was significantly decreased compared to TP2. However, in the intergroup comparison there was a significant difference in TP3 compared to TP2. In relation to SnE and SE, the SOD and CAT enzymes showed the most significant decrease in TP3 and the lowest decrease was observed in NSE, indicating that the values of these enzymes (CAT, SOD) were still significantly higher than the initial activities. It can be postulated that SOD and CAT not only play a fundamental, but also an indispensable role in the antioxidant protective capacity of biological systems against free radicals [34]. It seems that high activities of these enzymes in the recovery period are a factor to deal with this stress [35].

Souza et al. demonstrated that only a single acute exercise session is able to increase total antioxidant capacity in saliva samples [36,37] but the response of human salivary POX activity after smoking cigarettes showed that it could inactivate a high percentage of POX rapidly in vitro similar to in vivo conditions; coinciding with another study that observed this augmentation, whereas the reduction in POX correlated with the gaseous phase of cigarette smoke. In a study on mice exposed to cigarette smoke, it was shown that free radicals reduced the activity of SOD and CAT enzymes, resulting in an accumulation of H_2_O_2_ and lipid hydro-peroxidation. Moreover, the study showed a 60% decrease in POX activity in vivo; H_2_O_2_ is not removed from the oral cavity and causes oxidative damage [38,39]. These results are consistent with the findings of our study. POX plays a more crucial role of inhibiting the peroxidation process and therefore protects cells from oxidative stress [40]. For example, people who smoked more than 20 cigarettes per day significantly decreased salivary peroxidase activity for a long time, reducing the protective effect of peroxidase from the oral cavity against free radicals [39]. 

Consistent with the present study, Souza et al. showed in a study of salivary samples before and after three different exercise protocols on trained men, that after the exercise, CAT activity had increased significantly compared to pre-exercise [36]. It seems that training experience may express some kind of adaptations with the release of free radicals and increase the expression of enzymes in the respective genes which have higher antioxidant activity. Studies have shown that antioxidant activity in the body does not diminish after chronic and acute intense exercise to prevent the effects of increased free radicals [31,41,42]. Because exercise induces neutrophil and cytokine accumulation in damaged muscle, oxidative stress can be induced [37]. However, many studies suggest that in people who are undergoing chronic physical activity, adaptive and anti-oxidant defense systems increase [43,44]. Robertson et al. compared the antioxidant status of well-trained, moderate-activity, and untrained runners, and found that well-trained runners had a better antioxidant status than untrained subjects and that it had a significant correlation with the volume of the training, and those who have the highest oxygen consumption have higher antioxidant activity [45], which is in line with the results of this study. Bogdanska et al. in a study conducted on important salivary antioxidant found that the activity of the enzyme in smokers’ erythrocytes did not differ significantly from that of healthy subjects [46]. In the present study, the activity of this enzyme in NSE and NSnE was observed to be higher in comparison with SnE and SE.

A more severe decrease in resting baseline activities of CAT was observed in the SnE and SE compared to the other non-smoker groups. We also observed the inability to maintain the secreted enzyme after exposure to stressful conditions. This enzyme has the most important role in cleansing the cell membrane degradation induced by ROS and prevents over-degradation of these species by prolonging the time elapsed after exposure to stressful conditions [47,48] as in TP3, although it was observed that activities of this enzyme in the NSE were still relatively high compared to TP1, whilst in the aforementioned groups they had almost returned to resting activities.

Interestingly, it should be noted that antioxidant enzyme activity follows a cascade mechanism that reduces the activity of an enzyme and increases the oxidizing substances and ROS and reactive nitrogen species, which in turn augments the activity of other antioxidant enzymes. In the present study, which was performed on SnE and SE, decreased activity of both enzymes was observed at resting activities compared to NSnE and NSE. Ubiquinone radicals created in cigarette smoke can convert molecular oxygen into superoxide so that the high generation of this radical can reduce the activity of SOD in the saliva of people exposed to cigarette smoke due to the inactivation of oxidants in the smoke phase [46]. It appears that SnE and SE have lower SOD activities than the NSE and NSnE in antioxidant defense at TP1, in line with the results of Suriyaprom, et al. who demonstrated smokers had significantly lower SOD activities than non-smokers and indicated that smokers could have weakened antioxidant defense systems to modify the immune response [49].

Although immediately after exposure to stressful conditions or oxidative stress such as acute intense exercise in this study, activities of this variable increased similar to other groups, according to scientific principles, the most important role of enzymatic antioxidant defense is in post-exposure conditions.

The results of this study showed that in the first recovery period, after the stressful condition, the activities of this variable were significantly lower than the activities of the other two variables. The imbalance between the overproduction of free radicals and antioxidant ability to defend the body create conditions such as oxidative stress. Consistent with this view, Souza et al. and Suzuki et al. suggested that antioxidant and anti-inflammatory molecules are acting as a defense mechanism against the free radical generation (presupposed oxidative stress) induced by exercise [36,42]. These responses seemed to be overwhelming, inducing antioxidant and anti-inflammatory defense systems, and preventing exercise-induced oxidative stress [9,42]. However, this defense factor alone cannot completely neutralize the oxidative stress caused by pathological and physiological conditions [50]. It is also an important defense factor for faster adaptation and regeneration of ROS-induced cellular damage, especially in physiological and pathological conditions [51,52]. 

The two limitations of the present study could be the small statistical population in the groups and the lack of measurement of alpha-amylase activity and protein content in salivary.

## 5. Conclusions

To the best of our knowledge, the basic activities of defensive antioxidant enzymes are very important in coping with stressful situations that everybody faces on a daily basis (highly intense exercise, heat, cold, altitude, etc.); considering that, physiologically, the most important antioxidant defense role after exposure to these stresses is to stabilize the internal homeostasis, which is likely disturbed by this moderate-intensity exercise. Probably a healthy lifestyle and not smoking, along with exercise activities, could play an important role in antioxidant defense of the body in the conditions mentioned above.

The most important finding in this study was the between-group differences in TP1 and TP2 activities in the SE and SnE, and to a lesser extent in the NSnE compared to the NSE. In the present study, due to within-group and intergroup differences, the most positive effect of exercise and the negative effect of smoking was on POX, which is probably the most sensitive enzyme among the variables and the least negative effect was on SOD. The susceptibility of these enzymes to each other is likely to be different, and further research needs to be done in the field of statistics to confirm such changes.

## Figures and Tables

**Table 1:** Characteristics of individuals in groups.

Groups	Age (Years)	Height (m)	Weight (kg)	BMI (kg/m^2^)	Body Fat %	VO_2max_
NSnE	21.7 ± 1.9	1.78 ± 0.06	75.4 ± 4.45	23.84 ± 1.35	15.73 ± 1.28	42.1 ± 3.7
NSE	22.5 ± 1.6	1.77 ± 0.04	70.8 ± 3.90	22.41 ± 0.49	12.08 ± 0.88	55.9 ± 4.0
SnE	23.9 ± 1.5	1.76 ± 0.03	72.3 ± 6.58	23.25 ± 2.26	14.94 ± 2.38	40.8 ± 4.2
SE	23.6 ± 1.2	1.78 ± 0.02	69.9 ± 2.47	22.07 ± 0.88	12.71 ± 0.85	54.9 ± 4.2

BMI = body mass index; VO_2max_ (mL.kg^−1^.min^−1^) = maximum oxygen consumption; NSnE = non-smokers that did not exercise; NSE = non-smokers that did exercise; SnE = smokers that did not exercise; SE = smokers that did exercise.

**Table 2:** The analysis of the average daily caloric and nutrient intake.

Groups	NSnE	NSE	SnE	SE	MFT
Variable	M ± SD	M ± SD	M ± SD	M ± SD	*p*
**Calorie Intake (kcals/day)**	2667.80 ± 156.09	2733.27 ± 122.55	2657.23 ± 144.64	2616.07 ± 70.4	0.15
**Carbohydrate (g)**	377.88 ± 23.06	387 ± 17.47	377.97 ± 19.19	371.12 ± 10.71	0.16
**Protein (g)**	94.55 ± 5.61	96.25 ± 4.81	93.73 ± 6	92.48 ± 3.05	0.37
**Fat (g)**	86.45 ± 4.99	88.48 ± 4.10	86.29 ± 5.06	84.63 ± 2.31	0.26
**Vitamin C (mg)**	73.04 ± 2.7	71.86 ± 3.6	72.08 ± 4.10	72.23 ± 3.11	0.87
**Vitamin E (mg)**	16.07 ± 1.38	16.05 ± 1.25	15.57 ± 1.49	16.04 ± 1.13	0.35

M= mean; SD =standard deviation. NSnE = non-smokers that did not exercise; NSE = non-smokers that did exercise; SnE = smokers that did not exercise; SE = smokers that did exercise; MFT = Main Effect of Time.

**Table 3:** Changes in POX, CAT, and SOD enzyme activities among groups at three time points.

Enzymes		Groups	Mean Difference	95% CI for Difference		Bonferroni
		Comparison		Lower	Upper	*p*
**TP1**	**OX (U/mL)**	NSnE vs NSE	−0.007	−0.018	0.0031	0.341
		NSnE > SnE	0.016 ^#^	0.005	0.026	0.001 *
		NSnE > SE	0.012 ^#^	0.002	0.022	0.015 *
		NSE > SnE	0.023 ^#^	0.012	0.033	<0.001 *
		NSE > SE	0.019 ^#^	0.009	0.030	<0.001 *
		SnE vs SE	−0.003	−0.014	0.007	1
**TP2**	**POX (U/mL)**	NSnE < NSE	−0.031 ^#^	−0.043	−0.018	<0.001 *
		NSnE > SnE	0.019 ^#^	0.007	0.032	0.001 *
		NSnE > SE	0.011	−0.001	0.024	0.106
		NSE > SnE	0.050 ^#^	0.037	0.063	<0.001 *
		NSE > SE	0.042 ^#^	0.029	0.055	<0.001 *
		SnE vs SE	−0.008	−0.021	0.005	0.536
**TP3**	**POX (U/mL)**	NSnE < NSE	−0.018 ^#^	−0.030	−0.006	0.001 *
		NSnE > SnE	0.013 ^#^	0.001	0.025	0.026 *
		NSnE > SE	0.009	−0.003	0.021	0.253
		NSE > SnE	0.031 ^#^	0.019	0.043	<0.001 *
		NSE > SE	0.027 ^#^	0.015	0.039	<0.001 *
		SnE vs SE	−0.004	−0.016	0.008	1
**TP1**	**CAT (U/mL)**	NSnE vs NSE	−0.001	−0.004	0.002	1
		NSnE > SnE	0.003 ^#^	0.00	0.007	0.019 *
		NSnE > SE	0.004 ^#^	0.001	0.007	0.007 *
		NSE > SnE	0.004 ^#^	0.001	0.007	0.003 *
		NSE > SE	0.005 ^#^	0.002	0.008	0.001 *
		SnE vs SE	< 0.001 *	−0.003	0.003	1
**TP2**	**CAT (U/mL)**	NSnE < NSE	−0.009 ^#^	−0.015	−0.003	0.001 *
		NSnE vs SnE	−0.001	−0.008	0.005	1
		NSnE < SE	< 0.001 *	−0.006	0.006	1
		NSE > SnE	0.008 ^#^	0.002	0.014	0.005 *
		NSE > SE	0.009 ^#^	0.003	0.015	0.001 *
		SnE < SE	0.001	−0.005	0.008	1
**TP3**	**CAT (U/mL)**	NSnE vs NSE	−0.004	−0.009	0.00	0.056
		NSnE vs SnE	0.002	−0.002	0.007	0.907
		NSnE vs SE	0.00	0.004	0.005	1
		NSE > SnE	0.007 ^#^	0.002	0.011	0.001 *
		NSE > SE	0.005 ^#^	0.00	0.009	0.041 *
		SnE vs SE	−0.002	−0.007	0.002	1
**TP1**	**SOD (U/mL)**	NSnE vs NSE	−0.030	−0.096	0.037	1
		NSnE > SnE	0.129 ^#^	0.062	0.196	<0.001 *
		NSnE > SE	0.098 ^#^	0.032	0.165	0.001 *
		NSE > SnE	0.158 ^#^	0.091	0.225	<0.001 *
		NSE > SE	0.128 ^#^	0.061	0.195	<0.001 *
		SnE vs SE	−0.030	−0.097	0.037	1
**TP2**	**SOD (U/mL)**	NSnE < NSE	−0.109 ^#^	−0.190	−0.029	0.003 *
		NSnE > SnE	0.139 ^#^	0.059	0.019	<0.001 *
		NSnE vs SE	0.036	−0.044	0.016	1
		NSE > SnE	0.248 ^#^	0.168	0.329	<0.001 *
		NSE > SE	0.145 ^#^	0.065	0.226	<0.001 *
		SnE < SE	−0.103 ^#^	−0.183	−0.023	0.006 *
**TP3**	**SOD (U/mL)**	NSnE < NSE	−0.084 ^#^	−0.154	−0.014	0.011 *
		NSnE > SnE	0.132 ^#^	0.062	0.202	<0.001 *
		NSnE > SE	0.093 ^#^	0.023	0.163	0.004 *
		NSE > SnE	0.216 ^#^	0.146	0.286	<0.001 *
		NSE > SE	0.177 ^#^	0.107	0.247	<0.001 *
		SnE vs SE	−0.039	−0.109	0.031	0.776

CI = confidence interval, POX = peroxidase, CAT = catalase, SOD = superoxide dismutase, TP1 = time point from Pre-test, TP2 = time point immediately after the test, TP3 = time point one hour after the test; NSnE = non-smokers that did not exercise; NSE = non-smokers that did exercise; SnE = smokers that did not exercise; SE = smokers that did exercise. * Represents statistically significant differences among groups in time points, *p* < 0.05. ^#^ Represents mean difference with statistical significance at the 0.05 level.

## Data Availability

The data presented in this study are available on request from the corresponding author.
